# Molecular Approaches to Screen Bioactive Compounds from Endophytic Fungi

**DOI:** 10.3389/fmicb.2016.01774

**Published:** 2016-11-14

**Authors:** M. Vasundhara, Anil Kumar, M. Sudhakara Reddy

**Affiliations:** Department of Biotechnology, Thapar UniversityPatiala, India

**Keywords:** endophytic fungi, secondary metabolites, bioactive compounds, paclitaxel, podophyllotoxin, camptothecin, molecular markers

## Abstract

Endophytic fungi are capable of producing plant associated metabolites and their analogs with therapeutic value. In order to identify the potential endophytic isolates producing bioactive compounds, one need to screen all isolated endophytes, which may run into hundreds. Isolation of endophytic fungi is relatively a simple process; but screening of the isolated fungi for required metabolite production is a cumbersome process. Endophytic fungi producing plant associated metabolites may contain genes involved in the entire biosynthetic pathway(s). Therefore, ascertaining the presence of key enzymes of a particular biosynthetic pathway could serve as a molecular marker for screening of these endophytes to produce that metabolite. In absence of entire biosynthetic pathways in endophytic fungi, plant genes associated with that metabolic pathway could serve as markers. This review focuses on the impact of molecular approaches to screen the endophytic fungi for the production of bioactive compounds. An attempt has been made on screening of anticancer compounds like taxol (paclitaxel), podophyllotoxin, and camptothecin using molecular markers. The advantages of molecular approaches over conventional methods to screen endophytic fungi and also identification of endophytic fungi are discussed.

## Introduction

Endophytic fungi live in the tissues of plants without causing any symptoms of disease (Bacon and White, 2000) and the majority of these endophytes are fungi (Strobel and Daisy, 2003). Endophytic fungi are associated with the host plants, protect the host from pathogens, and at times may become opportunistic pathogens. Majority of endophytic fungi possess biosynthetic capabilities more than the associated host because of their long co-evolution and genetic recombination. Endophytic fungi have been considered as a source for novel natural bioactive compounds with potential application in medicine, agriculture, and food industry (Strobel et al., 1993; Suryanarayanan et al., 2009; Kharwar et al., 2011). Many endophytic fungi are capable of synthesizing various bioactive compounds that are used as therapeutic agents against numerous diseases (Suryanarayanan et al., 2009; Deshmukh et al., 2015).

The production of novel metabolites by fungal endophytes raises questions about the acquisition of the capacity to produce such complex metabolites by these organisms. The plant-endophyte co-evolution hypothesis suggests that it might be possible for endophytic fungi to assist the plant in chemical defense by producing these secondary metabolites (Ji et al., 2009). The possibility of acquisition of the capacity to produce these metabolites by endophytes from the host is supported by the fact that these endophytes harbor similar biosynthetic pathway and genes encoding enzymes catalyzing various steps as that of the host (Chandra, 2012). There are a few studies showing that the fungi isolated from the particular host produce active principle produced by that host (Stierle et al., 1993; Puri et al., 2005, 2006; Kusari et al., 2009, 2011; Nadeem et al., 2012; Garyali et al., 2013). The endophytic fungi producing such compounds have also shown the presence and expression of the similar homologous genes involved the biosynthesis of respective compounds in their host (Jennewein et al., 2001).

Although, each plant species is known to harbor many endophytic fungi, only a very minor fraction of them are able to produce important metabolites. This fraction has also been reported to belong to different taxa and is not confined to a particular taxonomic group. Therefore, in order to identify the potential isolates capable of producing a particular compound one needs to screen all isolated endophytes that usually runs into hundreds. Although isolation of endophytic fungi is relatively a simple process, screening of the isolated fungi for required metabolites is, however, a cumbersome process (Zhou et al., 2007; Xiong et al., 2013). Under such circumstances there is a possibility of missing some strains that are capable of producing these metabolites. Various procedures used for screening of this diverse group of organisms for the production of bioactive metabolites have been reviewed.

The present work provides an overview on the screening of endophytic fungi for production of the bioactive metabolites. The impacts of molecular approaches to screen the bioactive compounds from endophytic fungi are elaborated by including some of the anticancer compounds. The merits of molecular approaches over conventional methods to screen endophytic fungi, identify those using molecular tools and future perspectives are also discussed.

## Methods of screening endophytic fungi

Endophytes provide a broad variety of bioactive secondary metabolites with unique structure, including alkaloids, benzopyranones, chinones, flavonoids, phenolic acids, quinones, steroids, terpenoids, tetralones, xanthones, and others compounds (Tan and Zou, 2001). These bioactive metabolites have pharmacological activity with wide-ranging applications such as antibacterials, antifungal, antiviral, immunomodulators, antiparasitics, antioxidants, and anticancer agents (Wang et al., 2011; Zhao et al., 2011; Deshmukh et al., 2015; Vasundhara et al., 2016). Traditionally, various tools have been used for the screening of endophytic fungi starting from the testing of biological activities through bioassays leading to the purification, identification, and characterization of the bioactive molecules. Each of the isolated endophytic fungus is required to be cultured and then extracted with different organic solvents for the isolation of the metabolites. All the extracts are then processed through various stages such as testing for the biological activity using various activity specific bioassays, purification of molecules responsible for the tested biological activity (Stierle et al., 1993; Zhou et al., 2007; Bedair and Sumner, 2008; Aly et al., 2010; Garyali et al., 2013; Roopa et al., 2015). These bioactive compounds can be identified at the molecular levels by using various spectroscopic techniques coupled with precision chromatographic equipment. Chemical structure and related spectroscopic data regarding several secondary metabolites including bioactive compounds is available in various databases such as Human Metabolome database (HMDB) (Wishart et al., 2009), the METLIN database (Smith et al., 2005), and the Madison Metabolomics Consortium Database (MMDB) (Cui et al., 2008). These compounds can be identified by comparing their spectroscopic data with the available spectroscopic data in databases. Although, data and structure of large number of compounds is stored in these databases, yet for many unreported compounds, the data is not available in these databases and are identified using various spectroscopic techniques such as molecular ion mass spectrometry and fragmentation pattern (Kind and Fiehn, 2007; Böcker et al., 2009). Recently, tandem mass spectroscopy coupled with precision liquid chromatography systems are also used to generate such data from relatively less purified extracts (Sawada and Hirai, 2013). Further, data generated using nuclear magnetic resonance and infra-red spectroscopies also provide vital information about the structure of the unknown compounds, which help in elucidating the molecular structure of these compounds (Castro et al., 2010; van der Hooft et al., 2011). Although, such techniques serve as powerful tools for the identification of molecules in the extract and also for the characterization of new molecules, the screening of large number of isolated endophytic fungi through these processes is laborious and there is always a possibility of missing isolates with a capacity to produce novel metabolites. Thus, there is a need for developing an efficient procedure for the screening of large number of isolated endophytic fungi to identify the strains capable of producing specific novel pharmaceutically important compounds.

## Genome mining

The quest for the discovery of novel bioactive compounds has opened a new chapter with the availability of enormous genetic data. This information has been explored by mining the whole-genome sequence to identify biosynthetic pathways for undetected metabolites. To discover the new natural products and gene clusters for known metabolites, this information has been “fished out” of DNA libraries (Van and Shen, 2006). Genomes of filamentous fungi reveal that they contain far more gene clusters for secondary metabolite biosynthesis than estimated from the previously identified metabolites. These gene clusters encode enzymes for different classes of secondary metabolites such as non-ribosomal peptide synthetases, terpene synthases and polyketide synthases, known as “signature” genes/enzymes. These genes are presumed to be the founders of secondary metabolic gene clusters. They also contain genes for tailoring enzymes which modify the skeleton of secondary metabolites (For e.g., oxidoreductases, acyltransferases, glycosyltransferases, and methyltransferases) (Osbourn, 2010). Mining of genomes of *Aspergillus* spp., revealed the existence of about 40 cryptic biosynthetic gene clusters for secondary metabolites per genome. It has been reported that *A. nidulans* is capable of generating 32 polyketides, 14 non-ribosomal peptides and two indole alkaloids (Brakhage et al., 2008; Rank et al., 2010). The secondary metabolic gene clusters are silent under standard laboratory conditions in filamentous fungi, due to which no product can be formed. Brakhage and Schroeckh (2011) reviewed the strategies to activate silent gene clusters of fungal secondary metabolites. They showed that the majority of successful approaches to activate the gene clusters are based on the generation of gene “knock outs,” over expression of transcriptional factors, promoter exchange and other pleiotropic regulators. Other strategies such as epigenetics and simulation of the natural habitat of the same ecosystem will promote the activation of silent gene clusters and the production of novel metabolites in *A. nidulans*. They suggested that the simulation strategies play an important role to discover new bioactive compounds. Although the secondary metabolic pathways of many compounds are not well understood, the advancements in the areas of functional genomics and metabolomics, many genes of biosynthetic pathways of various secondary metabolites have been identified and characterized (Wankhede et al., 2013; Lau and Sattely, 2015).

Manipulation of the synthesis of bioactive compounds by endophytic fungi by genome mining will increase the yield and new derivatives with possible superior qualities. For the metabolites where the biosynthetic pathways are partly or fully known in one or more plant taxa, application of polymerase chain reaction help in elucidating the biosynthetic genes in the endophyte producing the same compound (Van and Shen, 2006; Kusari and Spiteller, 2011).

## Screening of endophytic fungi using molecular markers

Each step in a biosynthetic pathway is catalyzed by specific enzyme encoded by respective gene. Thus, for an organism to attain the capacity to produce a particular metabolite, it is necessary to acquire all the genes for encoding various enzymes catalyzing the production of that metabolite. Therefore, ascertaining the presence of key genes encoding important enzymes of that particular biosynthetic pathway could serve as a marker for the potential of these endophytes to produce those metabolites (Kusari and Spiteller, 2011). Various enzymes of biosynthetic pathway of paclitaxel (Taxol) and related taxanes are well characterized and genes encoding these enzymes are cloned (Kusari et al., 2013). It has been reported that fungi showing amplification of DNA fragments specific to genes involved in taxol biosynthesis namely, taxa-4(5),11(12)-diene synthase (*ts)*, debenzoyltaxane-2′-α-*O*-benzyol transferase (*dbat*) and the gene encoding final step in taxol biosynthetic pathway i.e., baccatin III 13-*O*-(3-amino-3-phenylpropanoyl) transferase (*bapt*) were able to produce taxol (Zhou et al., 2007; Zhang et al., 2008; Xiong et al., 2013). However, Garyali et al. (2013) reported that the fungal strains showing the amplification of only *ts* and *dbat* were found to be negative for taxol production. Hence, it is essential to select the appropriate genes/enzymes as markers for screening of endophytic fungi for production of bioactive compounds. Use of such initial screening is likely to reduce the time and improve the efficiency of screening. With less screening time and efforts, one may be able to quickly identify the important isolate from huge diversity of isolated fungal endophytes.

## Screening for bioactive compounds

### Paclitaxel

**Figure 1:**
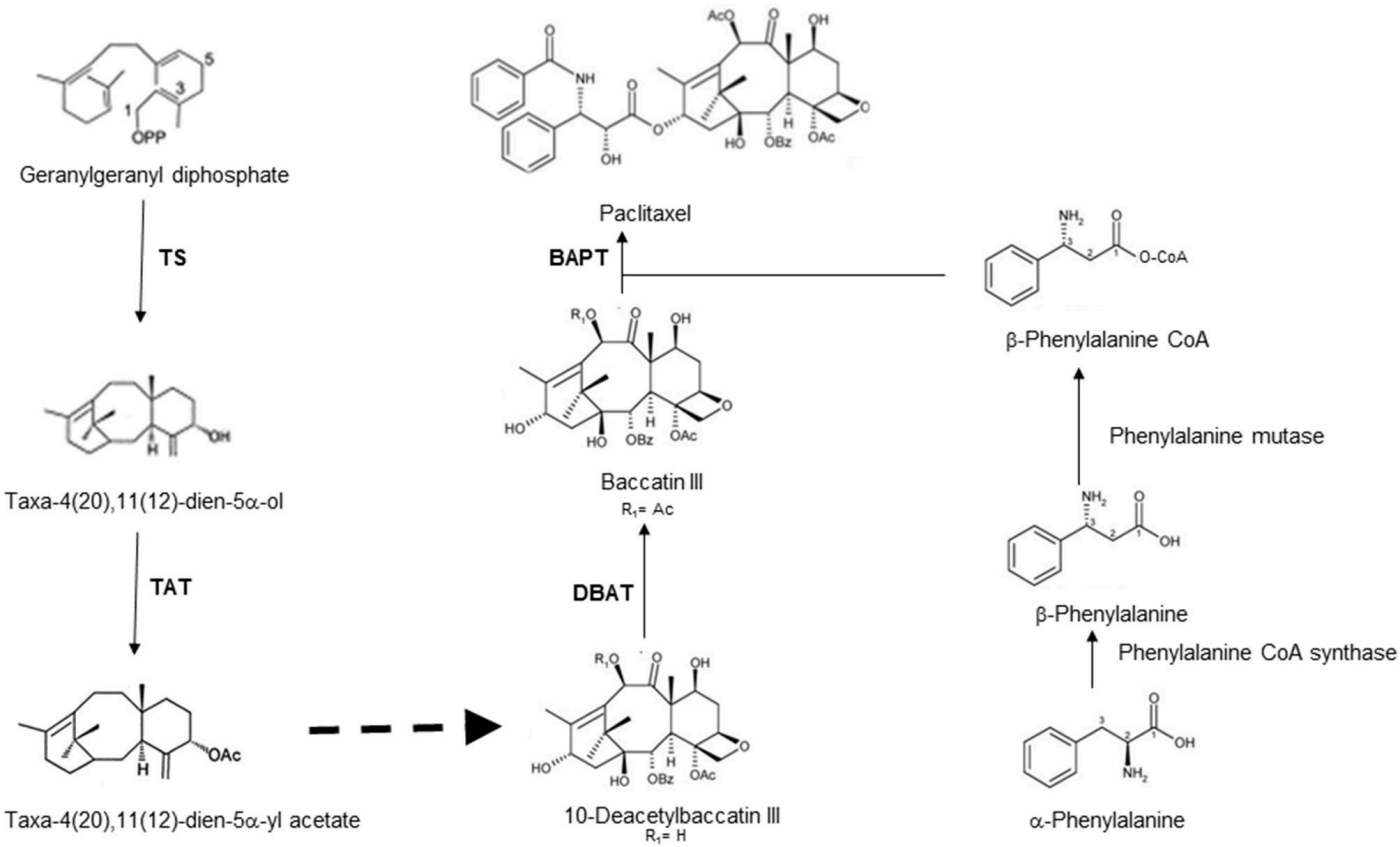
**Schematic representation of biosynthetic pathway leading to taxol along with corresponding enzymes catalyzing steps in taxol biosynthesis [TS: taxadiene synthase; TAT: taxe-4(20), 11(12)-diene-5α-ol-)-acetyltransferase; DBAT: 10-deacetylbaccatin III 10-*O*-acetyl-transferase; BAPT: baccatin III 13–*O*-(3-amino-phenylpropanoyl) transferase]**.

Paclitaxel (taxol) is a diterpenoid and extensively used against a range of cancer types. This compound is the world's first anticancer drug originally isolated from *Taxus brevifolia* (Pacific yew), and known to be present in all *Taxus* species (Strobel et al., 1996). Although, a complete chemical synthesis of taxol has been reported (Holton et al., 1994; Nicolaou et al., 1994), yet the method is highly uneconomical. At present, most taxol is prepared by semi-synthesis from baccatin III or 10-deacetyl baccatin, precursors of taxol, and these compounds also are isolated from yew trees (Collin, 2001). With all these efforts, the demand for the taxol far exceeds the supply. This gap in the demand and supply of taxol has sparked the efforts for the search of alternate sources for taxol. A ray of hope in this direction came with the discovery of endophytic fungi capable of producing taxol, which was isolated from the *Taxus* species (Stierle et al., 1993). Subsequently many workers reported the isolation of huge number of endophytic fungi from various plants and screened this large number of fungi to identify a few capable of producing taxol from yews. These endophytic fungi mostly belonging to ascomycetes and imperfect fungi that includes the genera *Pestalotia, Pestalotiopsis, Alternaria, Seimatoantlerium, Sporormia, Fusarium, Trichothecium, Tubercularia, Pithomyces, Monochaetia, Penicillium*, and *Truncatella* amongst others (Flores-Bustamante et al., 2010). Biosynthetic pathway of paclitaxel requires about 19 enzymatic steps with diterpenoid precursor geranylgeranyl diphosphate (GGPP) in yew trees (Hezari and Croteau, 1997). The first step involves the cyclization of GGPP to taxa-4(5),11(12)-diene by taxadiene synthase. The biosynthetic pathway of paclitaxel is represented in Figure 1.

**Figure 2:**
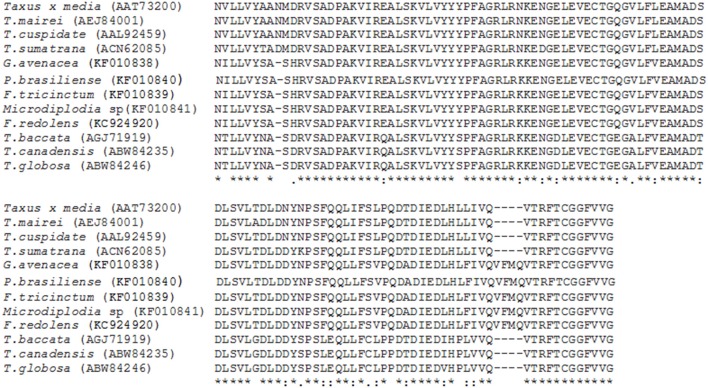
**Alignment of deduced amino acid sequences of BAPT from various species of *Taxus* and endophytic fungal isolates showing high level of sequence homology**.

The molecular basis of taxol biosynthesis in endophytic fungi is still not known. The screening of endophytic fungi for the production of paclitaxel using some of the biosynthetic genes as molecular markers are reported in Table 1. The nucleotide sequence of these genes and amino acid sequence of encoded enzymes of endophytic fungi showed high homology with related genes of *Taxus* species (Figure 2). The PCR amplification and cloning of genes of taxol biosynthetic pathway from different taxol producing endophytic fungi facilitated the potential alternative and sustainable source of taxol (Kusari et al., 2014). Therefore, PCR based molecular markers specific to taxol biosynthetic pathway genes could be effectively used for the screening of large number of isolated endophytic fungi.

**Table 1:** **Taxol synthesizing genes reported from endophytic fungi**.

**Fungus**	**Host**	**Accession No**.	**References**
**TAXADIENE SYNTHASE (TS)**			
*Mucor rouxianus*	*Taxus chinensis*	–	Zhou et al., 2007; Miao et al., 2009
*Fusarium solani*	*T. celebica*	HM113487	–
*Taxomyces andreanae*	*T. brevifolia*	–	Staniek et al., 2009
*Gibberella intermedia*	*Taxus x media*	KC337345	Xiong et al., 2013
**10-DEACETYLBACCATIN III-10-O-ACETYL TRANSFERASE (DBAT)**			
*Cladosporium cladosporoides*	*Taxus x media*	EU375527	Zhang et al., 2009a
*Fusarium solani*	*Taxuscelebica*	EF626531	–
*Aspergillus candidus*	*Taxus x media*	EU883596	Zhang et al., 2009b
*Lasiodiploidia theobromae*	*Sara caasoca*	KP136287	–
**BACCATIN III AMINOPHENYLPROPANOYL-13-O-TRANSFERASE (BAPT)**			
*Taxomyces andreanae*	*T.brevifolia*	–	Staniek et al., 2009
*Colletotrichum gloeosporioides*	*Taxus x media*	KC337344	Xiong et al., 2013
*Guignardia mangifera*	*Taxus x media*	KC337343	Xiong et al., 2013
*Fusarium redolens*	*T.baccata sub* sp. *wallichiana*	KC924919	Garyali et al., 2013
*Fusariun tricinctum*	*T. baccata sub* sp. *wallichiana*	KF010842	Garyali et al., 2014
*Fusarium avenaceum*	*T.baccata sub* sp. *wallichiana*	KF010843	Garyali et al., 2014
*Paraconiothyrium brasiliense*	*T. baccata sub* sp. *wallichiana*	KF010844	Garyali et al., 2014
*Microdiploidia* sp.	*T. baccata sub* sp. *wallichiana*	KF010845	Garyali et al., 2014
*Alternaria* sp.	*T. cuspidate*	GU323557	–

Genes of taxol biosynthetic pathway namely 10-deacetylbccatin-III-10-O-acetyl transferase (*dbat)* from *Clasdosporium cladosporiodes* showed 99% sequence similarity with the host plant *Taxus media* and 97% with same gene isolated from *T. wallichiana* (Yang et al., 2014). These findings point toward the possibility that the endophytic fungi have acquired the gene(s) for the synthesis of these novel compounds from their host by the process of horizontal gene transfer. On the other hand, Xiong et al. (2013) isolated three endophytic fungi from *T. media*, which are capable of producing taxol. The taxadiene synthase (*ts*) and *bapt* genes isolated from these fungi show very low levels of similarity with the corresponding genes from host i.e., *T. media* indicating that these fungi evolved these genes independently and have not acquired through horizontal gene transfer from the host.

### Podophyllotoxin

Podophyllotoxin is a lignan and is chemically converted to pharmaceutically important compounds namely teniposide, etoposide, etopophos, and other compounds. These compounds have high anticancer activity and are prescribed for the treatment of many types of cancers (Ekstrom et al., 1998; Holm et al., 1998; Ajani et al., 1999). These compounds are known to interact with topoisomerase II and inhibit the activity of this enzyme which is vital for DNA replication and cell division (Loike and Horwitz, 1976; Horwitz and Loike, 1977; Minocha and Long, 1984). The presence of podophyllotoxin and its various precursors have been reported from all the species of *Podophyllum*, a herbaceous perennial alpine rosette belonging to family berberidaceae (Haijun et al., 2004). Some of the other plant species which produce podophyllotoxin include *Linum flavum* (Broomhead and Dewick, 1990), *Juniperus verginiana* (Kupchan et al., 1965), *Hyptis verticillata* (Kuhnt et al., 1994). Due to over extraction and slow growing nature, *Podophyllum* plants have been listed among the endangered plant species (Chaurasia et al., 2012). Therefore, to maintain the sustained supply of podophyllotoxin for the preparation of anticancer molecules, there is an urgent need to search for the alternative sources. Among other alternative sources, endophytic fungi have been screened for their potential for the production of podophyllotoxin (Puri et al., 2006). Eyberger et al. (2006) reported the production of podphyllotoxin by two endophytic strains of *Phialocephala fortinii* isolated from *Podophyllum peltatum*. Kour et al. (2008) reported the production of podophyllotoxin by *Fusarium oxysporum* isolated from *Juniperus recurva*.

**Figure 3:**
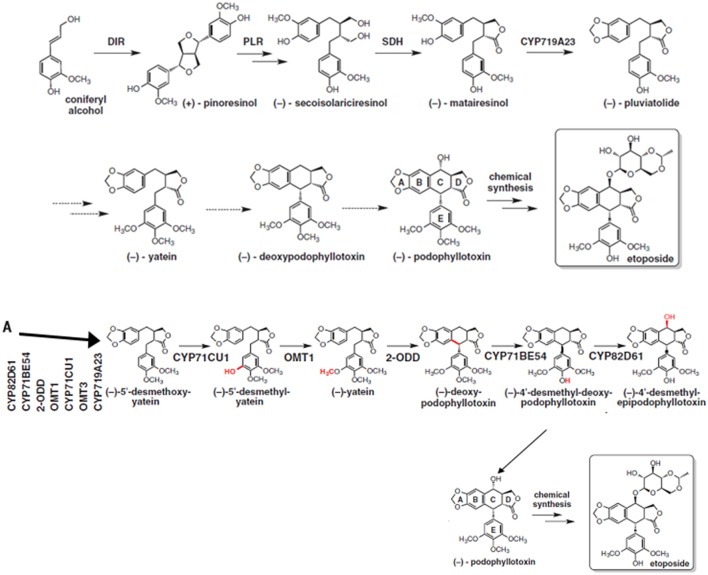
**Biosynthetic pathway of podophyllotoxin along with newly proposed steps (A)**, the steps with dotted arrows indicate the gaps that needs to be worked out in this pathway. From Lau and Sattely (2015). Reprinted with permission from AAAS.

Podophyllotoxin is synthesized through phenylpropanoid pathways; which is ubiquitously distributed among plant species and play important role in plant defense (Fukuda et al., 1985; Figgitt et al., 1989). Until recently, the knowledge about its biosynthetic pathway was fragmented and key enzymes and corresponding genes were not known (Kumar et al., 2015). A thorough knowledge of complete biosynthetic pathway would ease the access to podophyllotoxin and its natural derivatives which are difficult to produce synthetically (Kamal et al., 2015). Lau and Sattely (2015) have been able to fill the major gaps in the podophylltoxin biosynthetic pathway with the identification of six new enzymes catalyzing the key steps of podophyllotoxin production (Figure 3). A sequence of enzymes involved in podophyllotoxin biosynthetic pathway are dirigent protein (DIR), to coniferyl alcohol to (+)-pinocresol, which is converted by pinocresol-lariciresinol reductase (PLR). PLR is converted to (−)-secoisolariciresinol, which is further converted to (−)-matairesinol by sericoisolariciresinol dehydrogenase (SDH). This will further converted by CYP719A23 to (−)-pluviatolide. This is likely converted by Phex13114 (OMT1) to (−)-yatein, which is converted by Phex30848 (2-ODD) to (−)-deoxypodophyllotoxin (Lau and Sattely, 2015). The PCR based markers can be developed for some of the genes encoding these enzymes and used for the screening of large number of isolated fungal endophytes for podophyllotoxin. Such screening procedure will serve as an aid to screen the isolated endophytic fungi and also other organisms capable of producing podophyllotoxin.

### Camptothecin and related molecules

**Figure 4:**
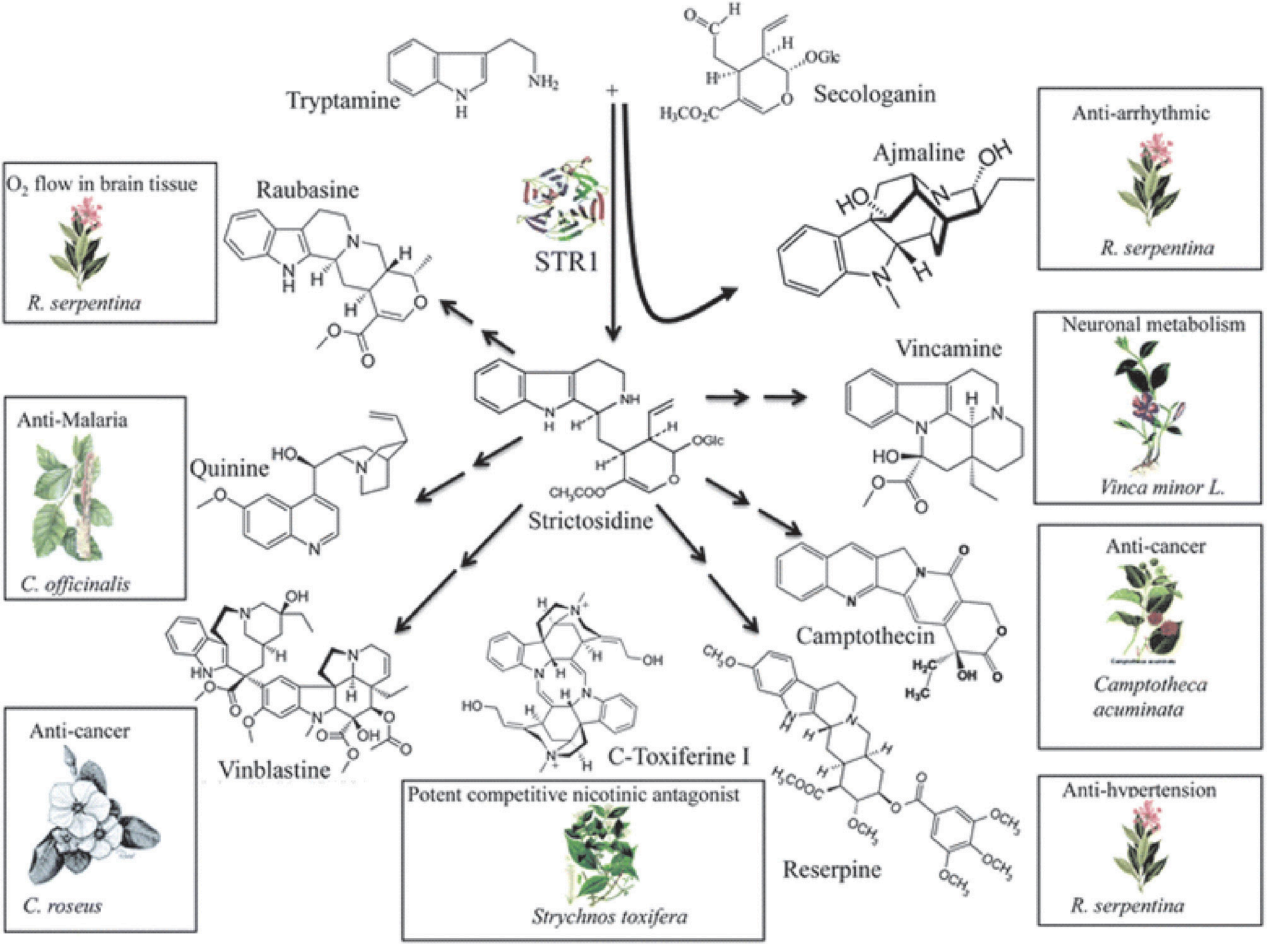
**Biosynthesis of strictosidine and its subsequent conversion to important alkaloids including anti-cancer compounds**. Reproduced from Panjikar et al. (2012) with permission of The Royal Society of Chemistry.

Camptothecin is a plant based alkaloid, which exhibits antitumor activity due to the inhibition of DNA topoisomerase I (Hsiang et al., 1985). Semi-synthetic water soluble analogs of camptothecin (Topotecan and Irinotecan) are prescribed for the treatment of tumors world over. The main precursor for the biosynthesis of camptothecin and many other important alkaloids including some important anti-cancer molecules is strictosidine, which is synthesized by the action of strictosidine synthase from tryptamine and secologanin (STR1) (Figure 4) (Panjikar et al., 2012). Figure 4 depicts the biosynthesis of strictosidine and its subsequent conversion to various important alkaloids with varied medicinal properties. Strictosidine is also a precursor for the synthesis of vinblastine, another important anticancer drug used for the treatment of different cancers. As strictosidine acts as a precursor for variety of alkaloids and detection of the gene encoding enzyme STR1 catalyzing its synthesis could provide significant information about this pathway. Gene encoding STR has been cloned and characterized from hairy root cultures of *Ophiorrhiza pumila* (Yamazaki et al., 2003). Although much of the information regarding the steps in-between strictosidine and camptothecin is not available, Cui et al. (2015) reported that the co-overexpression of geraniol-10-hydroxylase and strictosidine synthase increased camptothecin accumulation in *O. pumila* hairy root cultures.

*Fusarium solani* isolated from *Apodytes dimidiata* showed the production of 10-hydroxycamptothecin, 9-methoxycamptothecin, and camptothecin (Shweta et al., 2010). Kusari et al. (2009) reported the production of camptothecin and its analogs by an endophytic fungus from *Camptotheca acuminate*. Rehman et al. (2008) reported the production of camptothecin by an endophytic fungus *Neurospora* sp., isolated from *Nothapodytes foetida*. Camptothecin producing endophytic fungi, *Trichoderma atroviride* LY357, *Aspergillus* sp. LY355, and *Aspergillus* sp. LY341 were isolated from *C. acuminata* by Pu et al. (2013). Therefore, markers specific to geraniol-10-hydroxylase and strictosidine synthase can provide important information.

## Molecular characterization and identification of endophytic fungi

**Figure 5:**
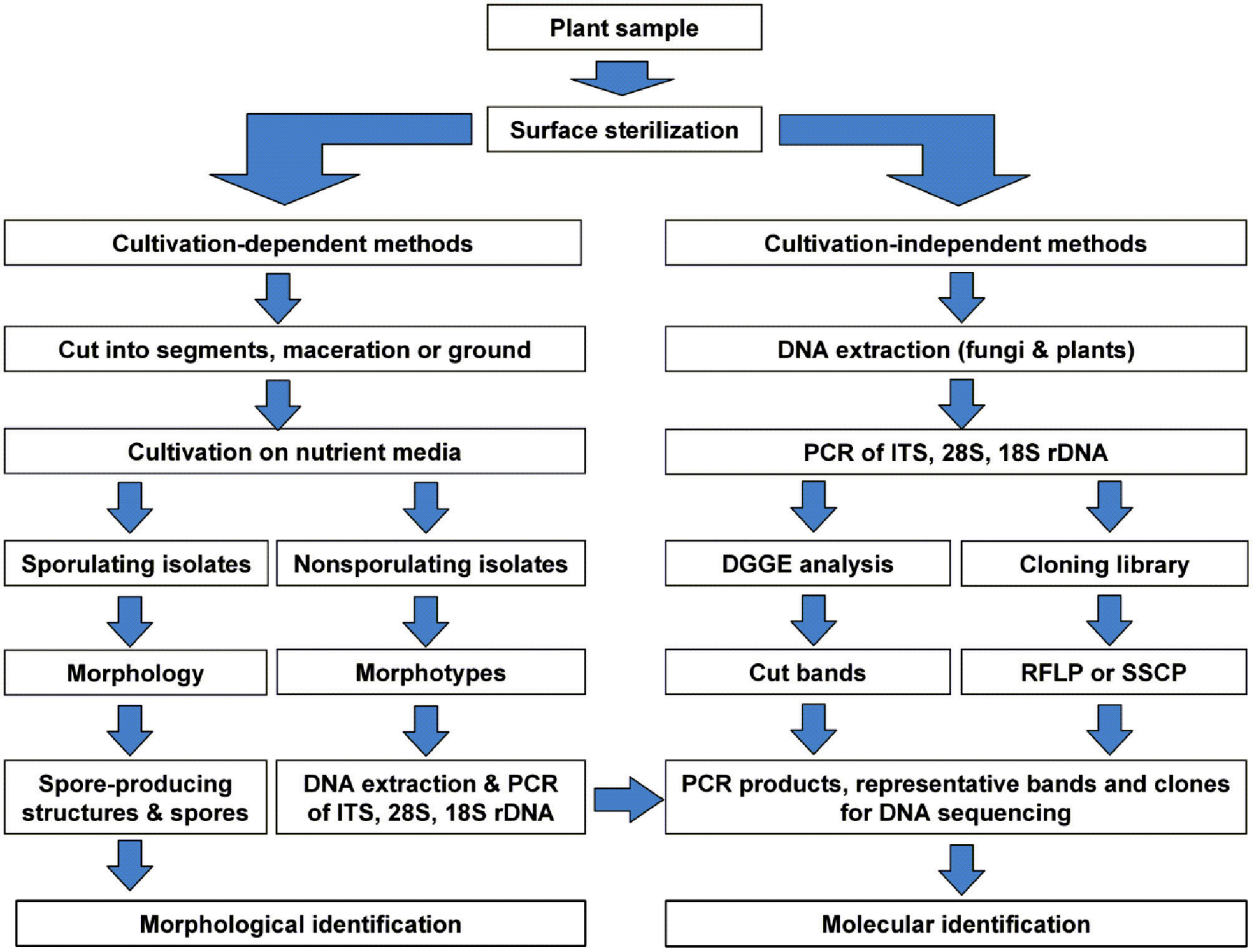
**Schematic diagram showing identification of the cultivable and non-cultivable endophytic fungal communities from a plant source**. Reprinted from Sun and Guo (2012). with permission from Mycological Society of China.

**Figure 6:**
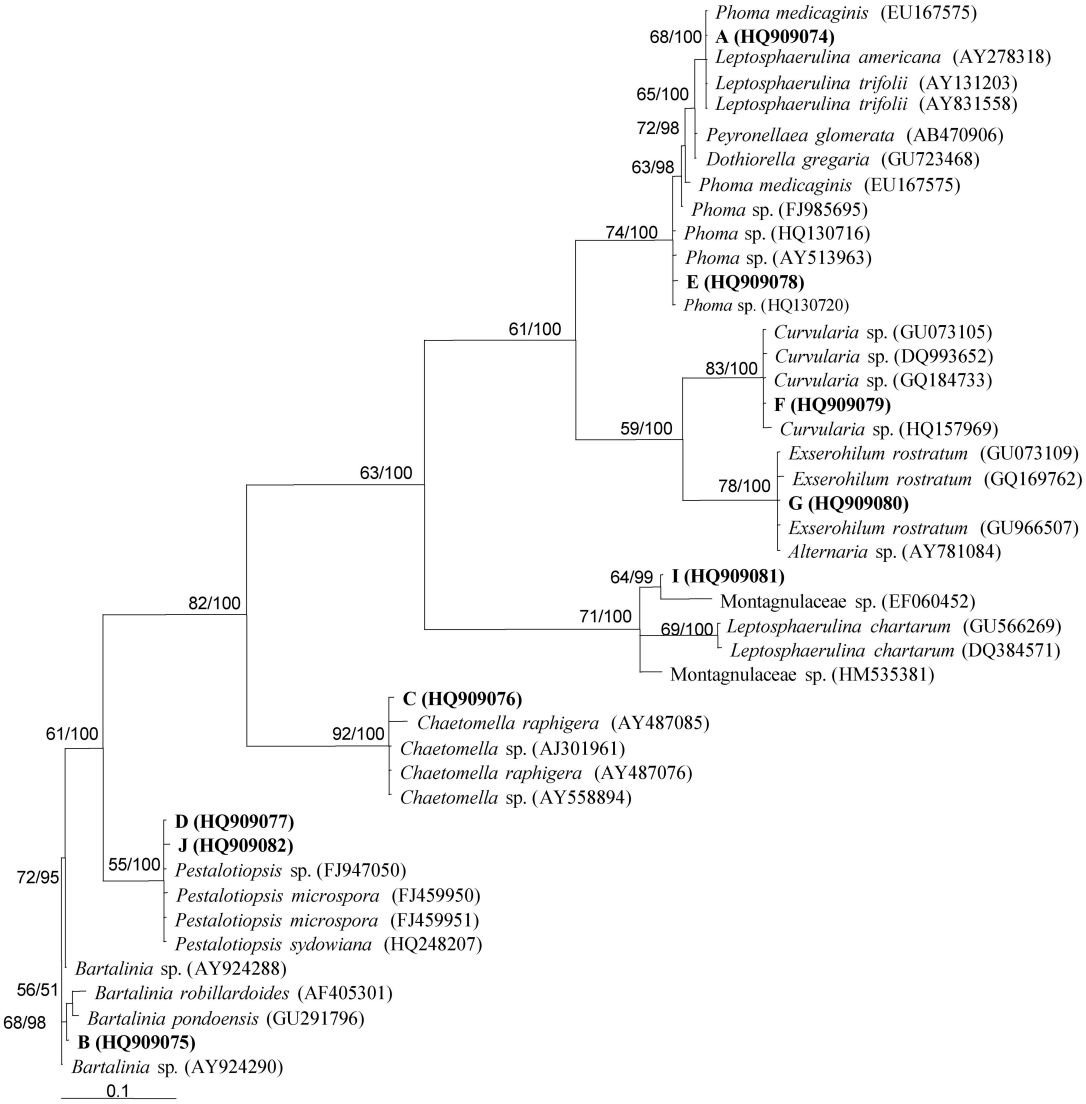
**Phylogenetic analysis of different endophytic fungi isolated from Western Ghats of India using parsimony analysis**. Parsimony bootstrap support (BS) and Bayesian PP values >50% are given at the internodes (BS/PP). Reprinted from Suryanarayanan et al. (2011). with permission from Elsevier.

Endophytic fungal communities comes from a broad range of fungal origins, which include Ascomycota, Basidiomycota, and Zygomycota. These fungal isolates can be identified based on their morphological characteristics if they sporulate on the media. Traditional classification of fungi heavily relies on reproductive structures, the non-sporulating fungi cannot be provided with taxonomic names (Sun and Guo, 2012). Application of molecular tools, such as DNA fingerprinting and sequencing methods (Figure 5), showed the potential to overcome the difficulties in traditional taxonomy for cultivable fungi. In endophytic fungi, 5.8S gene and flanking internal transcribed spacers (ITS1 and ITS2) of the rDNA, 18S and 28S rRNA genes have been employed in the identification of endophytic fungi. Pandey et al. (2003) identified different isolates of *Phyllosticta* that were isolated from different tropical tree species in India as *P. capitalensis* based on ITS sequence analysis. Morakotkarn et al. (2007) isolated 71 (of 257 strains) endophytic fungi from *Phyllostachy* and *Sasa* species and placed them into Sordariomycetes and Dothideomycetes based on 18S rRNA sequence analyses and further identified them into lower taxonomic levels based on ITS sequences. Endophytic fungi belong to Xylariaceae isolated from 22 tree species of a dry thorn forest and 27 tree species of a stunted montane evergreen forest of the Western Ghats in southern India were identified as *Xylaria* or *Nemania* species based on their ITS sequence analysis (Govindarajulu et al., 2013). Sun et al. (2011) clustered 221 non-sporulating endophyte strains into 56 morphotypes, and placed these morphotypes into 37 taxa based on ITS sequence similarity and phylogenetic analyses. Suryanarayanan et al. (2011) identified different endophytic fungi isolated from the Western Ghats of India based on ITS sequences and phylogenetic analysis (Figure 6).

Due to the limitations of traditional isolation procedures, it is highly probable that many endophytic fungi cannot be brought onto the culture. To overcome the potential technical bias, molecular approaches have been applied in the identification of endophytic fungi directly within the host tissues. This process involves (i) extraction of total genomic DNA from surface-sterilized plant tissues, (ii) amplification of DNA fragments (e.g., ITS, 28S and 18S genes) with fungal specific primers, (iii) denaturing gradient gel electrophoresis (DGGE) and excision of different bands (iv) cloning and sequencing of representative clones and identifying the sequences into various taxonomic levels based on phylogenetic analysis (Figure 5). High-throughput sequencing also serves as a powerful alternative to molecular studies of fungal community in natural environments. This technique has been successfully employed to study the fungal diversity in phyllosphere fungi (Jumpponen and Jones, 2009), mycorrhizal fungi (Dumbrell et al., 2011), and other natural environments. DNA barcoding systems is another technique employed to identify fungal species (Hebert et al., 2003). DNA barcode region used should be a single locus for all groups of organisms across all kingdoms. In endophytic fungi, ITS region is considered as the most widely used DNA barcode in molecular identification, despite some limitations in species distinction (Sun et al., 2011).

## Considerations

It has been reported that fungi showing amplification of DNA fragments specific to genes involved in taxol biosynthesis namely, *ts, dbat*, and *bapt* were able to produce taxol. However, it has been reported that the fungal strains showing the amplification of only *ts* and *dbat* were found to be negative for taxol production (Garyali et al. (2013). Hence, it is essential to select the appropriate genes/enzymes as markers for screening of endophytic fungi for production of bioactive compounds. Further, there are reports regarding the endophytic fungi producing important bioactive compounds which are not specific to host plants. Gangadevi and Muthumary (2008) reported the isolation of endophytic fungi *Colletotrichum gloeosporioides* capable of producing taxol from *Justicia gendarussa*, a plant not known for taxol production. Such exceptional cases need to be studied in detail to clarify the possibilities of acquiring these genes of biosynthetic pathways by endophytic fungi. Also use of gene specific primers will help in screening and identifying the bioactive compounds produced by endophytic fungi which are not specific to host plant.

## Conclusions

Endophytic fungi are important sources of therapeutically active compounds. Driven by the success of molecular screening of endophytic fungi capable of producing taxol, it seem rational to apply this technology for the compound specific screening of large number of isolated endophytic fungi. The possibility of using such procedures is high in case of novel compounds including anticancer compounds for which either complete or partial biosynthetic pathways are known. If the biosynthetic pathways are not known for some of the metabolites, plant genes associated with that metabolic pathway could be used as markers to screen the endophytic fungi.

## Author contributions

MV: Contributed in writing the manuscript (except podophyllotoxin and taxol portion). AK: Contributed writing the podophyllotoxin and taxol portion of the manuscript. MR: Contributed overall compilation and editing of the manuscript.

### Conflict of interest statement

The authors declare that the research was conducted in the absence of any commercial or financial relationships that could be construed as a potential conflict of interest.
